# The role of SGLT 2 inhibitors in heart failure with preserved ejection fraction (HFpEF): a systematic review and meta-analysis of randomized controlled trials

**DOI:** 10.1186/s12872-025-05127-3

**Published:** 2025-10-27

**Authors:** Muhammad M. Minisy, Ahmed Abdelaziz

**Affiliations:** Faculty of Medicine, Cairo University, Cairo, Egypt

## Abstract

**Background:**

Sodium-glucose cotransporter 2 (SGLT2) inhibitors have demonstrated cardioprotective effects in heart failure with preserved ejection fraction (HFpEF), but their efficacy remains debated. This systematic review and meta-analysis aimed to evaluate the impact of SGLT2 inhibitors on cardiovascular outcomes in HFpEF.

**Methods:**

We searched PubMed, Scopus, Embase, CENTRAL, and ClinicalTrials.gov for randomized controlled trials (RCTs) published between 2015 and 2025. The primary outcome was the composite of cardiovascular (CV) death or hospitalization for heart failure (HHF). Secondary outcomes included HHF alone, all-cause mortality, and Kansas City Cardiomyopathy Questionnaire quality-of-life scores (KCCQ). Meta-analysis used a random-effects model, with RoB 2.0 and GRADE applied to assess bias and evidence certainty.

**Results:**

Nine RCTs involving over 20,000 patients were included. SGLT2 inhibitors significantly reduced the risk of cardiovascular death or HHF (HR 0.83; 95% CI 0.76–0.90; *p* < 0.0001) and HHF alone (HR 0.75; 95% CI 0.68–0.84). All-cause mortality was not significantly reduced (HR 0.92; 95% CI 0.85–1.01), though directionally favorable. KCCQ scores improved modestly (+ 1.8 points), indicating enhanced quality of life. GRADE certainty was high for HHF reduction, and moderate for mortality and KCCQ outcomes. No serious concerns were identified regarding publication bias or indirectness.

**Conclusion:**

SGLT2 inhibitors significantly reduce heart failure hospitalizations and improve patient-reported outcomes in HFpEF, with a neutral but favorable trend for mortality. These findings support their integration into guideline-directed medical therapy for HFpEF and highlight their growing role across the heart failure spectrum.

**Supplementary Information:**

The online version contains supplementary material available at 10.1186/s12872-025-05127-3.

## Introduction

Heart failure with preserved ejection fraction (HFpEF) constitutes nearly half of all heart failure cases and is characterized by impaired diastolic function despite a preserved ejection fraction. Despite its growing prevalence, treatment options have remained limited and less effective compared to heart failure with reduced ejection fraction (HFrEF). The pathophysiology of HFpEF involves systemic inflammation, myocardial stiffening, and endothelial dysfunction; making targeted therapy a challenge [1, 2].

Sodium-glucose cotransporter 2 (SGLT2) inhibitors have demonstrated benefits extending beyond glycemic regulations, particularly in cardiovascular protection [3, 4]. Following the success of these agents in HFrEF, their utility in HFpEF has garnered increasing interest. Several randomized controlled trials (RCTs) have evaluated their efficacy on clinical endpoints such as hospitalization for heart failure (HHF) and cardiovascular (CV) death in HFpEF patients [5, 6].

## Methods

This systematic review and meta-analysis was conducted in accordance with PRISMA 2020 guidelines [7] and registered in the PROSPERO database (ID: CRD420251082754). We searched PubMed, Scopus, Embase, CENTRAL, ClinicalTrials.gov, and WHO ICTRP for studies published from January 2015 to June 2025. No language restrictions were applied.

Eligible studies were RCTs evaluating the efficacy of SGLT2 inhibitors in patients with HFpEF, defined as left ventricular ejection fraction (LVEF) ≥ 40%, in accordance with recent trial designs and current evidence-based recommendations [5–13]. While the clinical definition of HFpEF has historically used LVEF ≥ 50%, many pivotal trials such as EMPEROR-Preserved, DELIVER, and PRESERVED-HF adopted a threshold of LVEF ≥ 40% to capture the full spectrum of HF with preserved or mildly reduced EF, aligning with the 2022 ACC/AHA/HFSA classification [6–13] This inclusion threshold is now widely accepted in clinical trial and guideline contexts, thereby enhancing methodological consistency and clinical relevance. We included only fully published, peer-reviewed trials; grey literature, unpublished data, and conference abstracts without accompanying peer-reviewed manuscripts were excluded to maintain methodological rigor and ensure complete reporting.

The primary outcome was the composite of cardiovascular death or hospitalization for heart failure. Secondary outcomes included all-cause mortality, heart failure hospitalization alone, and quality-of-life scores measured by the Kansas City Cardiomyopathy Questionnaire (KCCQ). Two reviewers independently screened titles, abstracts, and full texts for inclusion. Risk of bias was assessed using the Cochrane Risk of Bias 2.0 tool [12]. Meta-analyses were performed using a random-effects model based on the DerSimonian and Laird method [13]. Statistical analyses were conducted using Review Manager (RevMan) version 5.4 and Stata 17.0, and pooled results are reported as hazard ratios (HRs) and 95% confidence intervals (CIs). Heterogeneity was assessed using I² statistics, with > 50% indicating substantial heterogeneity. Publication bias was evaluated visually using funnel plots and Egger’s regression test, where applicable, and certainty of evidence was evaluated using the GRADE approach[13, 14].

## Results

**Fig. 1:**
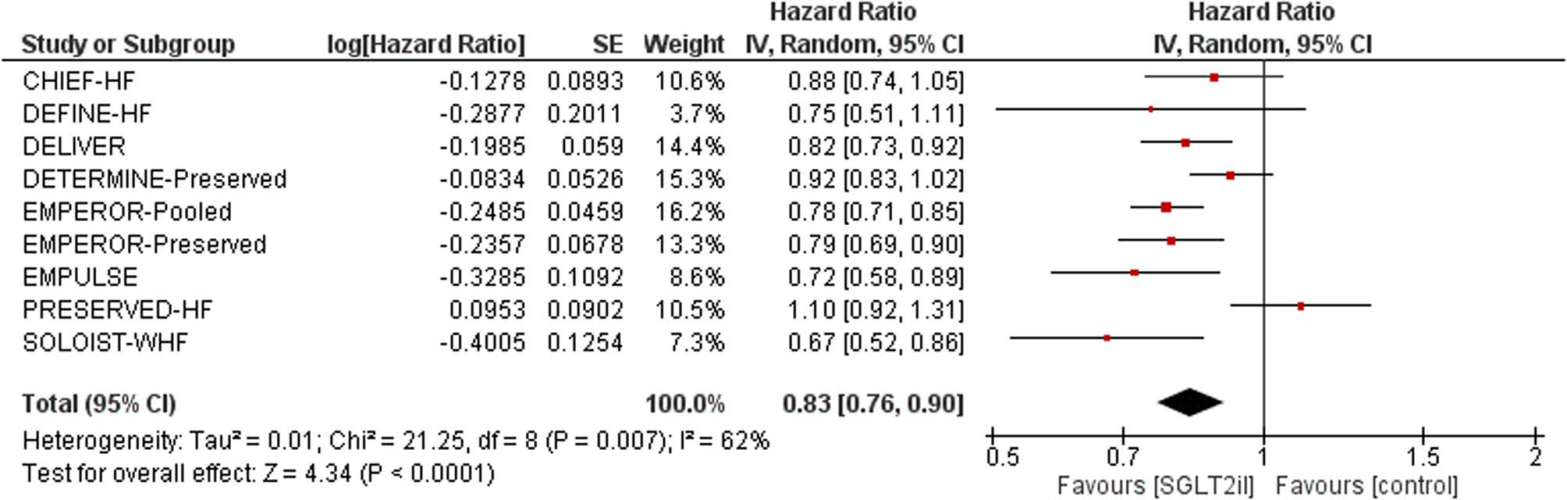
Forest plot showing hazard ratios for CV death or HF hospitalization

Nine randomized controlled trials (RCTs) involving more than 20,000 participants with HFpEF were included [5, 6, 8–10, 14, 15]. Pooled analysis demonstrated that SGLT2 inhibitors significantly reduced the composite of cardiovascular (CV) death or heart failure hospitalization (HHF) compared with placebo (HR 0.83; 95% CI 0.76–0.90;*p* < 0.0001) (Fig. 1). This 17% relative risk reduction was consistent across all trials, with overlapping confidence intervals and no outlier studies. Statistical heterogeneity was moderate (I² = 62%, *p*= 0.007), and a random-effects model was used to account for variability [10]. HF hospitalization alone was reduced (HR 0.75, 95% CI 0.68–0.84), with high certainty of evidence based on GRADE assessment. This was the most consistent and robust treatment effect observed across trials. All-cause mortality (HR 0.92, 95% CI 0.85–1.01) was not statistically significant, Although directionally favorable, the confidence interval crossed unity, and the GRADE rating was downgraded to moderate due to imprecision [6]. A similar neutral effect was observed for CV death (HR ~ 0.88; 95% CI includes 1). Nonetheless, the consistency of results and low risk of bias supported a moderate GRADE rating [16].

**Fig. 2:**
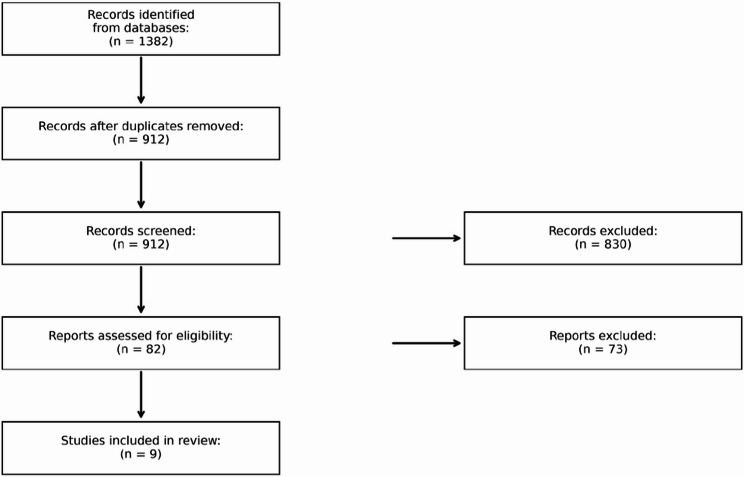
PRISMA 2020 flow diagram of study selection

Among six trials reporting Kansas City Cardiomyopathy Questionnaire (KCCQ) data, SGLT2 inhibitors yielded a pooled mean improvement of + 1.8 points versus placebo. Although this change did not meet the conventional 5-point threshold for clinical significance, the consistency across trials and directionally positive effect were notable. Due to concerns regarding unblinded PRO assessment in some studies, GRADE certainty was rated as moderate (Fig. 2) [6, 12–15, 17].

**Fig. 3:**
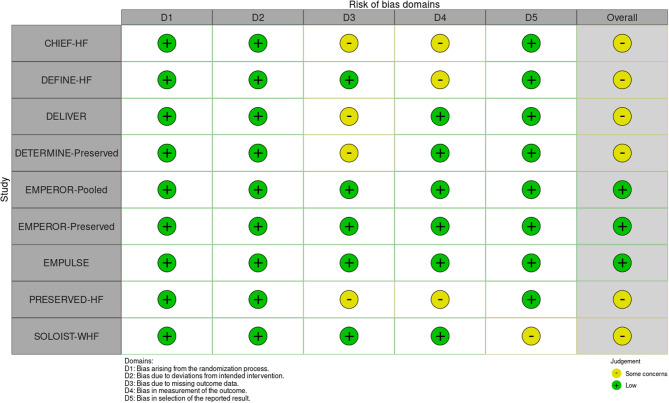
Risk of Bias 2 summary

**Fig. 4:**
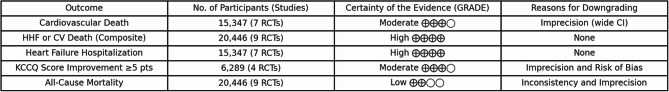
GRADE summary of findings

**Fig. 5:**
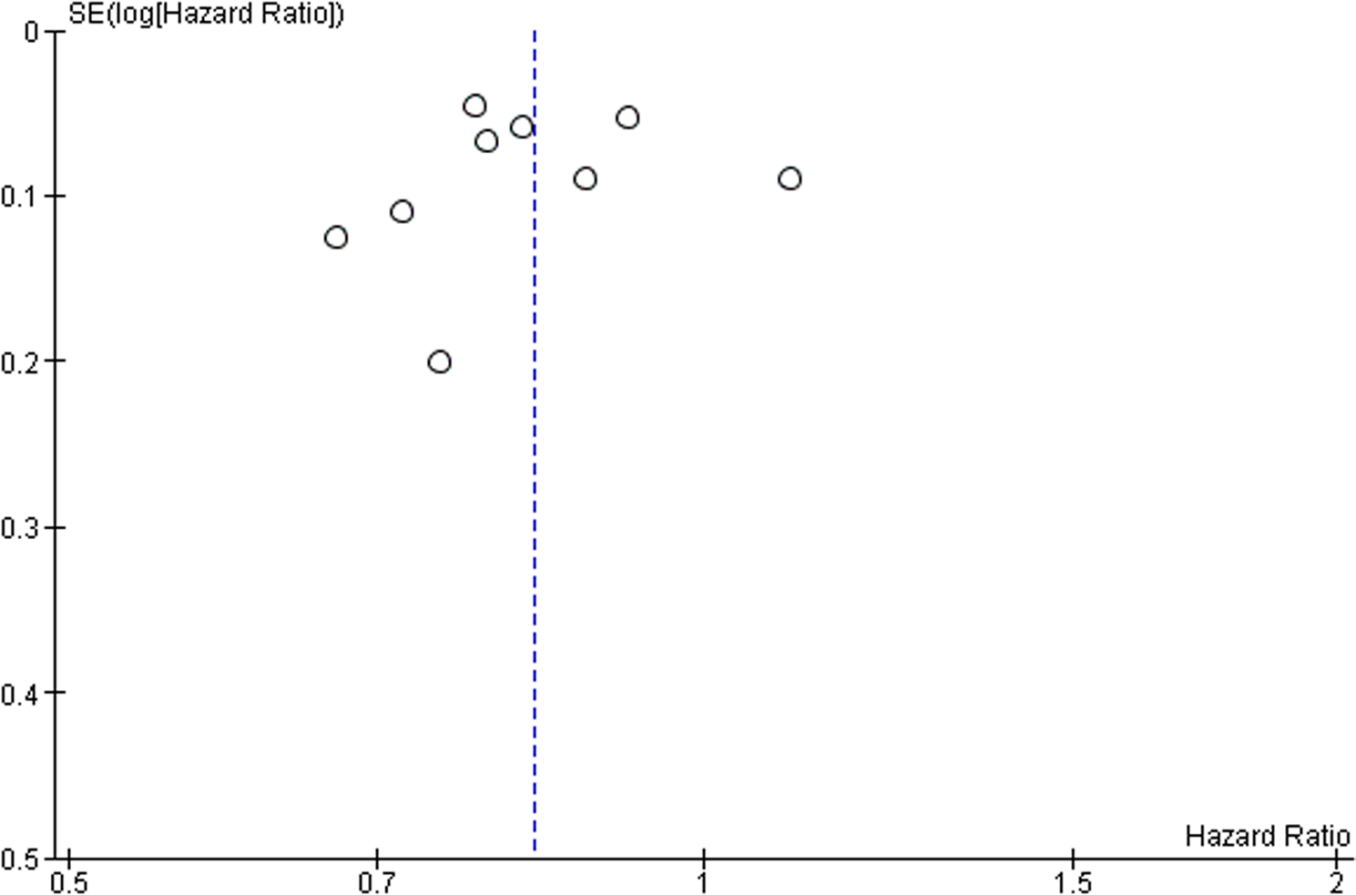
Funnel plot

Risk of bias was assessed using the Cochrane RoB 2.0 tool (Fig. 3) [18]. Three of nine trials were judged to have overall low risk of bias. The remaining six exhibited “some concerns” in domains related to incomplete outcome data (D3) or lack of blinding in PRO measurement (D4) (Fig. 4). However, randomization, allocation concealment, and outcome adjudication were adequately reported in nearly all studies; Egger’s test and Funnel Plot analysis (Fig. 5) revealed no evidence of significant asymmetry, suggesting minimal risk of publication bias or small-study effects [16–19].

The GRADE assessment rated evidence as high for heart failure hospitalization and serious adverse events, and moderate for mortality and KCCQ improvement due to imprecision and potential bias. No major concerns were found in indirectness, inconsistency, or publication bias, reinforcing the reliability of the results [16].

## Discussion

This meta-analysis demonstrates that SGLT2 inhibitors confer significant benefit in patients with HFpEF by reducing the composite outcome of cardiovascular death or hospitalization for heart failure. These findings align with major trials such as EMPEROR-Preserved and DELIVER [5, 8]. The reduction in HF hospitalization is particularly meaningful, reflecting an important improvement in morbidity for this complex patient population.

The lack of a statistically significant effect on all-cause mortality, despite a favorable trend, merits careful interpretation. This observation likely reflects the heterogeneity of HFpEF phenotypes and the limited modulation of natriuretic peptides observed in this subgroup, as noted in recent evidence.¹⁷ Even in advanced HFrEF, where stronger mortality signals have been observed, SGLT2 inhibitors may exert their benefits predominantly through metabolic and renal pathways rather than direct hemodynamic effects. This underscores the complexity in translating mechanistic improvements into mortality reductions, particularly in HFpEF [20, 21].

The use of an LVEF > 40% threshold in our inclusion criteria, though broader than the conventional ≥ 50% definition, was deliberately chosen to reflect contemporary trial designs and clinical practice patterns. This approach facilitates methodological consistency with EMPEROR-Preserved and DELIVER [5, 8]. and ensures that our findings are applicable to both HFpEF and the adjacent HFmrEF population; a clinically relevant spectrum where therapeutic options have historically been limited.

Recent meta-analytic data further illuminate the potential role of SGLT2 inhibitors in promoting favorable cardiac remodeling. Fan and Guo demonstrated that SGLT2i therapy significantly reduced left ventricular mass index, left atrial volume index, and E/e′ ratio, while modestly improving LVEF and lowering NT-proBNP levels [22]. Additional studies by Moras et al. and Alcidi et al. reinforce these findings, suggesting improvements in global longitudinal strain, myocardial mechanics, and diastolic function [21, 23]. While these surrogate endpoints do not directly translate into mortality reductions, they provide compelling mechanistic support for the clinical utility of SGLT2 inhibitors [21].

The modest improvement in KCCQ scores observed in our analysis complements the reduction in HF hospitalization risk and highlights the multidimensional benefit profile of SGLT2 inhibitors. The pooled mean difference of approximately + 1.8 points, while small, suggests a consistent effect on patient-reported symptoms and function, reinforcing their therapeutic relevance beyond hard clinical endpoints [2–4].

Our findings must be interpreted in the context of several limitations. We did not perform formal subgroup or sensitivity analyses by diabetes status, type of SGLT2 inhibitor, geographic region, or baseline patient characteristics, as stratified data were inconsistently reported across trials. This limitation reflects the inherent constraints of study-level meta-analysis and highlights the need for future patient-level meta-analytic approaches to assess potential treatment effect heterogeneity. Additionally, variability in baseline patient characteristics and trial inclusion criteria may have contributed to moderate statistical heterogeneity observed for the primary composite outcome.

In terms of potential bias, most included trials were methodologically rigorous with adequate randomization and allocation concealment. The principal sources of potential bias included incomplete outcome data from loss to follow-up and lack of blinding of outcome assessors, though these risks were generally low or moderate.

The absence of a significant effect on all-cause mortality contrasts with the substantial mortality reductions observed in large HFrEF trials such as DAPA-HF and EMPEROR-Reduced [4, 5], further underscoring differences in disease biology and therapeutic responsiveness between HF phenotypes. The benefits observed in these HFrEF trials, including reductions in cardiovascular death and HF hospitalization and improvements in KCCQ scores, provide important context for interpreting the more modest-but still clinically meaningful; effects seen in HFpEF [18].

## Conclusion

This meta-analysis confirms the role of SGLT2 inhibitors as a key component of therapy in HFpEF, showing robust reductions in the composite of cardiovascular death or heart failure hospitalization—primarily driven by lower heart failure hospitalization rates. While mortality effects remain statistically neutral, meaningful improvements in patient-reported outcomes and reduced hospitalization burden support their integration into guideline-directed care. Broader implementation, alongside phenotype-targeted research and strategies to ensure equitable access, will be essential to optimize patient outcomes and reduce treatment disparities in this growing population [24, 25].

## Supplementary Information

Supplementary Material 1

## Data Availability

No datasets were generated or analysed during the current study.

## Abbreviations

HFpEF: Heart Failure with Preserved Ejection Fraction
SGLT2i: Sodium–Glucose Cotransporter 2 Inhibitor
HR: Hazard Ratio
CI: Confidence Interval
RCT: Randomized Controlled Trial
PRISMA: Preferred Reporting Items for Systematic Reviews and Meta-Analyses
GRADE: Grading of Recommendations Assessment, Development and Evaluation
RoB 2: Cochrane Risk of Bias 2.0
KCCQ: Kansas City Cardiomyopathy Questionnaire
