# Supplementary material for: The role of SGLT 2 inhibitors in heart failure with preserved ejection fraction (HFpEF): a systematic review and meta-analysis of randomized controlled trials
Source: BMC Cardiovasc Disord. 2025 Oct 27;25:765. doi: 10.1186/s12872-025-05127-3 (PMC12557931; doi:10.1186/s12872-025-05127-3)
Supplement: Supplementary file 1 — Supplementary Material 1 [file 12872_2025_5127_MOESM1_ESM.pdf]

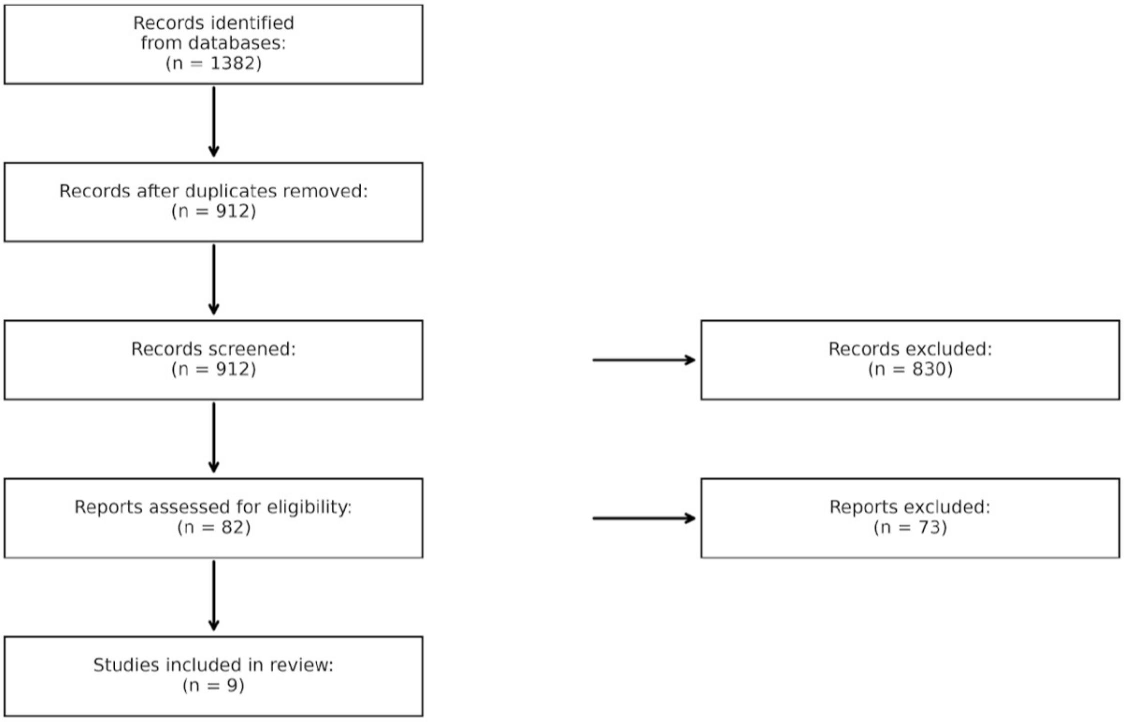
Records identified
from databases:
(n = 1382)
Records after duplicates removed:
(n = 912)
Records screened:
(n = 912)
Records excluded:
(n = 830)
Reports assessed for eligibility:
(n = 82)
Reports excluded:
(n = 73)
Studies included in review:
(n = 9)

Study or Subgroup	log[Hazard Ratio]	SE	Weight	Hazard Ratio IV, Random, 95% CI
CHIEF-HF	-0.1278	0.0893	10.6%	0.88 [0.74, 1.05]
DEFINE-HF	-0.2877	0.2011	3.7%	0.75 [0.51, 1.11]
DELIVER	-0.1985	0.059	14.4%	0.82 [0.73, 0.92]
DETERMINE-Preserved	-0.0834	0.0526	15.3%	0.92 [0.83, 1.02]
EMPEROR-Pooled	-0.2485	0.0459	16.2%	0.78 [0.71, 0.85]
EMPEROR-Preserved	-0.2357	0.0678	13.3%	0.79 [0.69, 0.90]
EMPULSE	-0.3285	0.1092	8.6%	0.72 [0.58, 0.89]
PRESERVED-HF	0.0953	0.0902	10.5%	1.10 [0.92, 1.31]
SOLOIST-WHF	-0.4005	0.1254	7.3%	0.67 [0.52, 0.86]
**Total (95% CI)**			**100.0%**	**0.83 [0.76, 0.90]**

Heterogeneity: $Tau^2 = 0.01$; $Chi^2 = 21.25$, df = 8 (P = 0.007); $I^2 = 62\%$
Test for overall effect: Z = 4.34 (P < 0.0001)

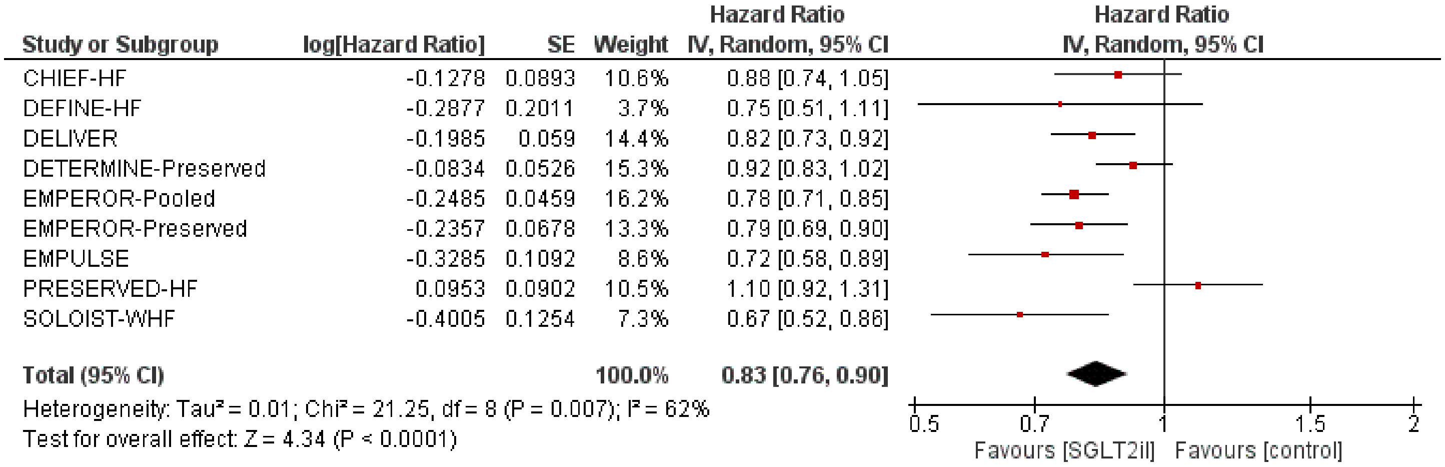

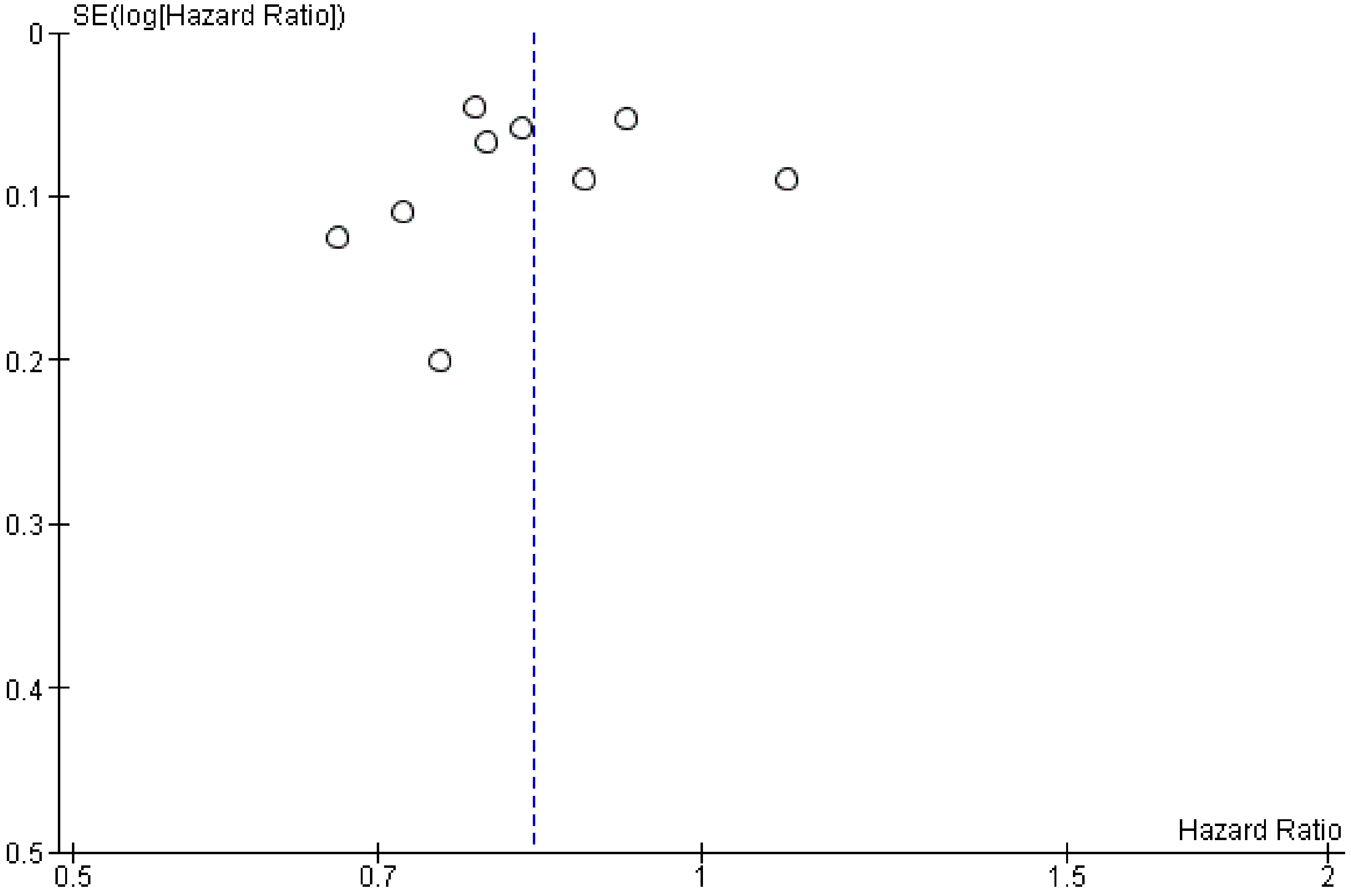
SE(log[Hazard Ratio])
0
0.1
0.2
0.3
0.4
0.5
Hazard Ratio
0.5
0.7
1
1.5
2

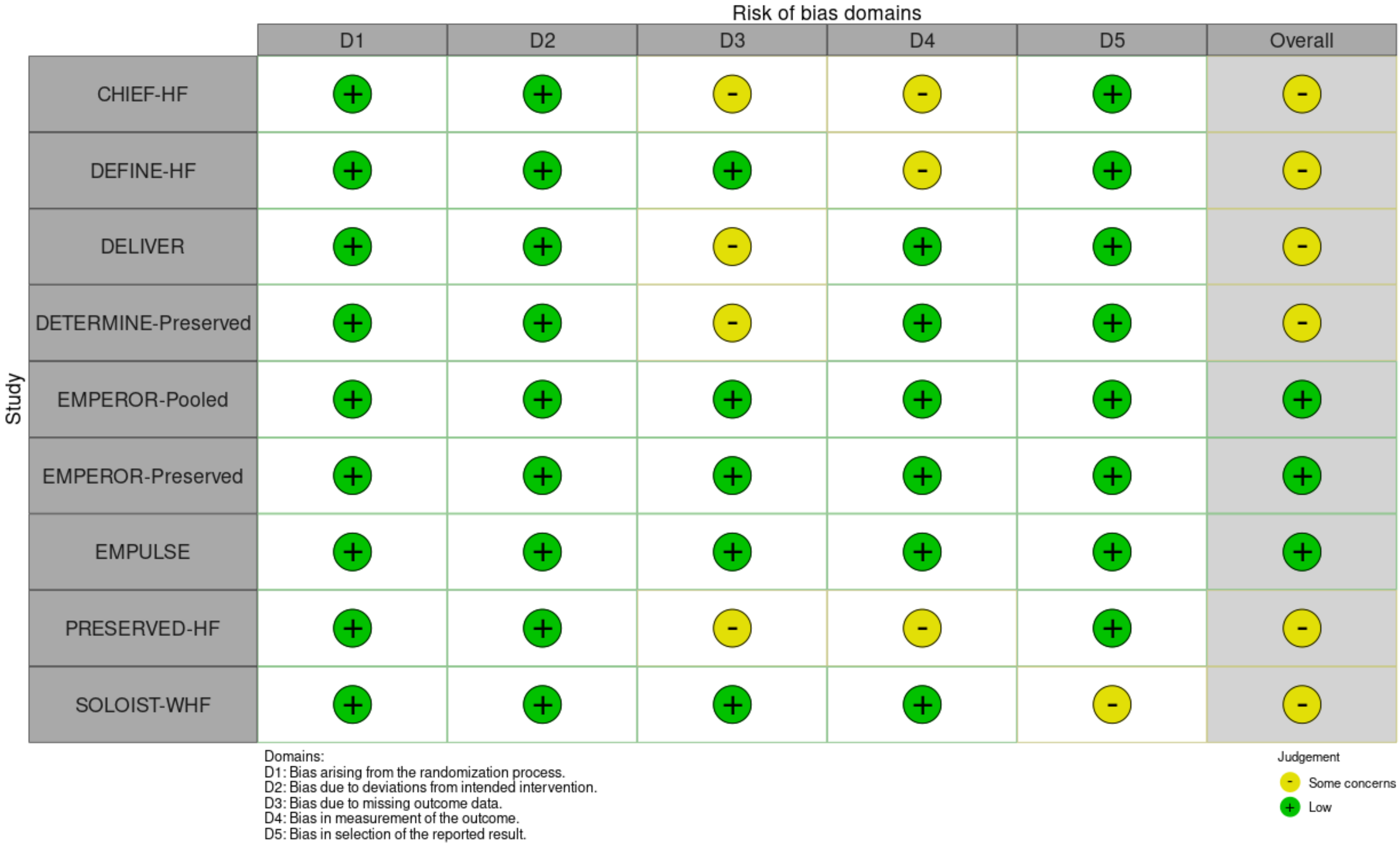
Risk of bias domains
D1
D2
D3
D4
D5
Overall
Study
CHIEF-HF
DEFINE-HF
DELIVER
DETERMINE-Preserved
EMPEROR-Pooled
EMPEROR-Preserved
EMPULSE
PRESERVED-HF
SOLOIST-WHF
Domains:
D1: Bias arising from the randomization process.
D2: Bias due to deviations from intended intervention.
D3: Bias due to missing outcome data.
D4: Bias in measurement of the outcome.
D5: Bias in selection of the reported result.
Judgement
Some concerns
Low

Outcome	No. of Participants (Studies	Certainty	Risk of Bias	Inconsistency	Indirectness	Imprecision	Publication Bias
Cardiovascular Death	20,245 (9 RCTs)	Moderate	Not serious	Not serious	Not serious	Not serious	Undetected
Heart Failure Hospitalizatior	20,000+ (9 RCTs)	High	Not serious	Not serious	Not serious	Not serious	Undetected
All-Cause Mortality	19,500+ (8 RCTs)	Moderate	Not serious	Not serious	Not serious	Serious (CI crosses 1)	Undetected
‹CCQ Improvement (≥5 poin	10,000+ (6 RCTs)	Moderate	Serious (some unblinded PRC	Not serious	Not serious	Not serious	Undetected
Serious Adverse Events	20,000+ (9 RCTs)	High	Not serious	Not serious	Not serious	Not serious	Undetected
